# Optical brush: Imaging through permuted probes

**DOI:** 10.1038/srep20217

**Published:** 2016-02-12

**Authors:** Barmak Heshmat, Ik Hyun Lee, Ramesh Raskar

**Affiliations:** 1 Media Lab, Massachusetts Institute of Technology, 75 Amherst St., Cambridge, MA, 02139, USA

## Abstract

The combination of computational techniques and ultrafast imaging have enabled sensing through unconventional settings such as around corners, and through diffusive media. We exploit time of flight (ToF) measurements to enable a flexible interface for imaging through permuted set of fibers. The fibers are randomly distributed in the scene and are packed on the camera end, thus making a brush-like structure. The scene is illuminated by two off-axis optical pulses. Temporal signatures of fiber tips in the scene are used to localize each fiber. Finally, by combining the position and measured intensity of each fiber, the original input is reconstructed. Unlike conventional fiber bundles with packed set of fibers that are limited by a narrow field of view (FOV), lack of flexibility, and extended coaxial precalibration, the proposed optical brush is flexible and uses off-axis calibration method based on ToF. The enabled brush form can couple to other types of ToF imaging systems. This can impact probe-based applications such as, endoscopy, tomography, and industrial imaging and sensing.

The introduction of fiber bundles was a turning point for sensing and imaging applications in harsh and industrial environments. The fiber bundles are flexible, immune to EM noise, and can tolerate extreme temperatures (e.g. up to 1500 °C in case of ruby fibers) and extreme pressures^1^,^2^,^3^. Coherent fiber bundles have been used as image guides in many endoscopy^4^,^5^ and microscopy applications^6^,^7^,^8^ (Fig. 1a). The fibers of a coherent fiber bundle have a constant arrangement along the axis of the fibers; therefore, any image that is fed into one end is received at the other end without any permutation or distortion. Such fiber bundles are necessary, especially in smaller environments where a camera cannot fit^9^. For industrial applications, longer coherent fiber bundles become too expensive and too rigid to be used; Levy *et al.* suggested using incoherent fiber bundles^10^ (ICFB) and calibrating them before use. By this calibration, the shuffled or permuted fibers can be sorted, or deshuffled, at the receiving end, and the image can be reconstructed computationally after it passes through permuted fibers.

This concept received more attention recently as more sophisticated binary codes can be used to deshuffle and precalibrate the fibers efficiently with a projector^4^,^11^,^12^,^13^ (Fig. 1b). Calibration by differential binary codes^4^, gray codes^13^, and fringe-based addressing^11^ was shown to be suitable for a packed incoherent fiber bundle that has fixed permutation. This system, however, is limited to a rigid cylindrical form factor. The second major drawback is the necessity for a high-resolution display or projector, which must be positioned accurately in front and coaxial with the bundle. This precludes extension of the method to dynamic recalibration in industrial environments and unreachable geometries.

Unfortunately, because all the fibers are bundled together, the field of view (FOV) is also usually narrow with a fixed form factor. Therefore, increasing the numerical aperture of a fiber bundle based imaging system has always been of interest^5^,^14^. Additionally, the physical dimensions of the bundles are just not small enough to see through more challenging porous media such as lung bronchioles or porous sponge-like forms.

Unlike packed fiber bundles, where fibers are right next to each other on both ends of the bundle, an optical brush (an open-ended incoherent fiber bundle) has a deformable form factor; potentially capable of penetrating into porous geometries and sensing in liquid turbid media. An optical brush opens up the bundled fibers on the scene end, randomly distributes them into the object space, and then transfers the light from these random points of the scene to different pixels on the sensor. This is as if each pixel of the camera sensor were receiving light from a random position in the scene (Fig. 1c). Because of the gap between the pixels, the brush is capable of sampling a much larger area compared to a packed bundle.

While a brush-like form factor is widespread for robust and large volume sensory in nature (e.g. sea plants), to the best of our knowledge, it has not been exploited in modern imaging and sensing systems. This is because the mentioned advantages come with major challenges, such as lower resolution and loss of spatial information for each sensor. Imaging through a swarm of sensors (Fig. 1d) based on sensor network theories^15^, might address some of these challenges for meso-scale subjects^16^. However, for micro scales the fabrication of electronic sensor networks is extremely demanding.

Recently, imaging research has experienced a paradigm shift with the emergence of advanced ultrafast^17^,^18^, time-of-flight (ToF)^17^,^19^,^20^,^21^ and computational^22^,^23^,^24^,^25^ imaging techniques. Such techniques usually use either direct or indirect measurement of ToF for a pulse or a sequence of temporal codes^24^. Based on these time signatures the shape of an object can be reconstructed even in exotic settings when the object is hidden around the corner or behind diffusive layers^19^,^20^. The combination of time-based imaging along with computation have also provided other strong contributions in high speed photocytometry^17^ and microscopy^22^.

In this work, we use a pulsed ToF technique^19^ for calibration of an optical brush to reconstruct the image of a scene through a randomly positioned set of fibers. Here, instead of using ToF to reveal 3D depth information, our method uses this parameter to change the physical form of the imaging or sensing interface for a second camera. This enabling perspective on ToF parameter can be combined with emerging and preexisting ToF techniques such as continuous wave (CW) ToF^24^, sequentially time all-optical mapping^26^ coherent interferometric depth imaging techniques^27^,^28^ and pulsed ToF methods^20^,^29^. Such change in physical form directly affects acquisition capability of these systems that are appealing for biomedical imaging^17^, photophysics^30^, and industrial sensing^19^. We experimentally and theoretically evaluate our technique by reconstruction of multiple images and comparing the results with coaxial raster scanning of individual scene points. Our analysis shows that an optical brush increases the field of view quadratically with increase in the variance of the fiber spatial distribution; this is at the expense of decrease in SNR. Finally, our technique does not require a coaxial calibration, offers a flexible field of view and is intrinsically multi-spectral. Therefore, it has significant potential for endoscopy, imaging in turbid media^31^,^32^,^33^,^34^,^35^ and near-field batch probing^8^,^36^.

## Results

**Figure 2:**
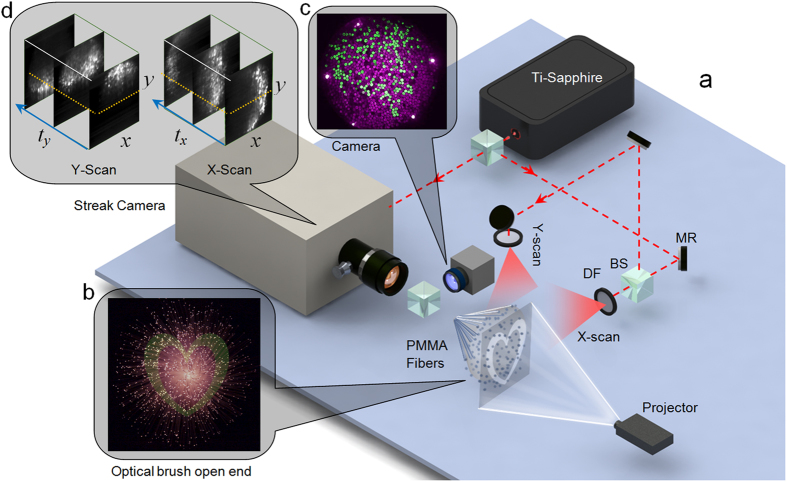
Experimental setup. (**a**) Setup consists of a streak camera triggered by Ti-Sapphire pulses and an ordinary camera placed on the close-end of the optical brush. The other end is fed with a synthetic scene via a projector. BS stands for beam splitter and DF stands for diffuser. (**b**) Front view of the open-end of the optical brush with image of the heart projected on to the fibers. The infrared pulses are propagating in off-axis (perpendicular) with the plane that the fibers are distributed in. (**c**) Shuffled output of the brush seen by an ordinary camera. (**d**) Streak camera output. Each *x-t* slice is a streak image.

The optical brush consists of 1,100 equally spaced, multi-mode, PMMA fibers (with 300 μm diameters). The bundle is packed on the camera end and is open on the scene end thus creating a brush-like form. The experimental setup is depicted in Fig. 2a. A streak camera (Hamamtsu C5680) and a visible-range CCD camera image the back (the close-end) of the optical brush simultaneously. A Ti-Sapphire laser beam (400 mW at 780 nm with 80 MHz repetition rate) is split and diffused With two thin polycarbonate diffusers to generate a vertical (X-scan) and horizontal (Y-scan) set of sweeping pulses. The same laser is also triggering the streak camera.

The two diffusers are placed 40 cm apart from the axis of the optical brush so that the horizontal and vertical scanning pulses have an approximately planar wavefront while passing by the bristles. The average power that is received by each fiber is roughly about 0.5 μW. The power spatial profile is nonuniform; it reduces about 3 folds as the pulses propagate across the brush plane and further away from the distanced diffusers. A projector is used to make a synthetic 2D scene for the brush. For example, the shape of heart is projected to the open-end of the optical brush (Fig. 2b). Since the fibers are randomly distributed in the 2D scene plane the camera sees a lossy permuted or “shuffled” image of the heart as in Fig. 2c. Therefore, a map between input and output positions of the fibers is needed to reconstruct the original image at the input-end (open-end). To obtain this map, a set of two *x-y-t* streak data cubes is recorded with the streak camera at the 780 nm wavelength (Fig. 2d). Because the fibers have equal length, the pulse propagation times inside the fibers are almost equal within the 2 ps time resolution of the streak camera. Therefore, the time that the pulse is received in the streak image for each fiber at the close-end (or output-end facing the cameras) correlates directly to the position of that fiber at the open-end (or input-end in the scene). For instance, since all the fibers are equal in length, a fiber that outputs the pulsed signal later in time by the sweeping X-scan pulse (propagating from right to left) should be also positioned further away to the left. Finally, to have a benchmark for comparison, the projector is used to localize each fiber by raster scanning the scene pixel by pixel.

**Figure 3:**
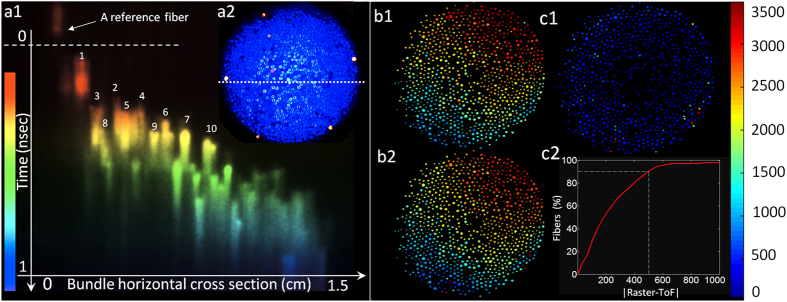
Deshuffling procedure and error. (**a1**) A sample *x-t* streak image for X-scan, the fibers can be sorted in time to reveal the X coordinates of the fibers on the open-end of the brush. Color bar on the left is indicative of the pseudo-color of the time axis. (**a2**) A sample image captured at the back of the brush by an ordinary camera for raster scan. One of the scene pixels is turned “on” in red while the other pixels are blue in X-Y space. (**b1**,**b2**) Reference and ToF-based shuffling map respectively. (**c1**) Difference between reference shuffling map and obtained ToF shuffling map. Color bar on the right represents the address value or address value difference for each fiber. (**c2**) Percentage of fibers that result in similar addresses based on both methods. Horizontal axis is similarity threshold and vertical axis is fiber percentage.

The permutation (or shuffling) map is a map between close-end or output-end coordinates (*x-y*) and the open-end or input-end location of each fiber in (X-Y) coordinates. The ToF and raster deshuffling processes are different as shown in Fig. 3. The permutation map is found by a set of streak images in the case of ToF technique. Figure 3a1 shows a sample recorded streak image for X-scan. This is an *x-t* slice recorded at a given *y* of the close-end cross section of the optical brush (dotted white line in Fig. 3a2). Figure 3a1 shows that by simply sorting the bright spots in time, the fiber positions can be recovered for a given horizontal cross section *y*. The first ten fibers are color-coded and labeled in Fig. 3a1 to demonstrate this procedure. The time window of the streak image (the vertical axis) is ~1 ns and the horizontal axis is 1.5 cm that corresponds to the diameter of the brush at the close-end facing the camera. By recording a streak (*x-t*) image for each *y,* the data cube is completed. By sorting out the entire peak signals from each fiber in time, X-Y position of each fiber on the open-end is found. Two set of such data cube is necessary to recover both X and Y of each fiber on the open-end of the brush. X-scan and Y-scan data cubes are measured sequentially and individually. By mapping the location information of each fiber onto the close-end image the shuffling map is obtained as in Fig. 3b1,b2.

The coaxial raster scanning with the projector provides a reference shuffling map (Fig. 3b1). To generate the reference shuffling map, the individual pixels of the scene were turned “on” one by one, and an image was captured by the visible range camera for each pixel position in X-Y space. Fig. 3a2 shows a sample output of one of the raster scan positions. As it can be seen only one of the fibers is red (on) while all the rest are blue (off). This fiber is the *x-y* representation of the “on” pixel in the X-Y plane, and therefore, by raster scanning the entire X-Y space the reference shuffling map is completed.

There are five bright fibers on the circumference of the close-end cross section as in Fig. 3a2; these are the reference fibers used as features for correctly registering the streak output data cube with the camera output. The five reference fibers are set in a way to light up always earlier than the rest of the fibers in the streak images. A reference fiber is visible (shown by white arrow) in the streak image (Fig. 3a1). The narrow time slot before the dashed line (the first 100 rows of the streak image from the top) is reserved for the five reference fibers.

The reference raster scan on the open-end divides the X-Y scene into 60 horizontal and 80 vertical lines; starting by the value “1” (dark blue) assigned to the position (1, 1) on the top left and ending by the value “4800” (red) assigned to the position (60, 80). The raster is done vertically; for example, the first column is scanned from (1, 1) to (60, 1) and then pixel (1, 2) is turned “on”. As in Fig. 3b1,b2, the red end is on the top right corner of the map which correctly indicates that the whole brush is twisted counter clockwise roughly about 90 degrees along its axis. Other than this main twist, it is notable that the fibers are randomly positioned relative to neighboring fibers.

**Figure 4:**
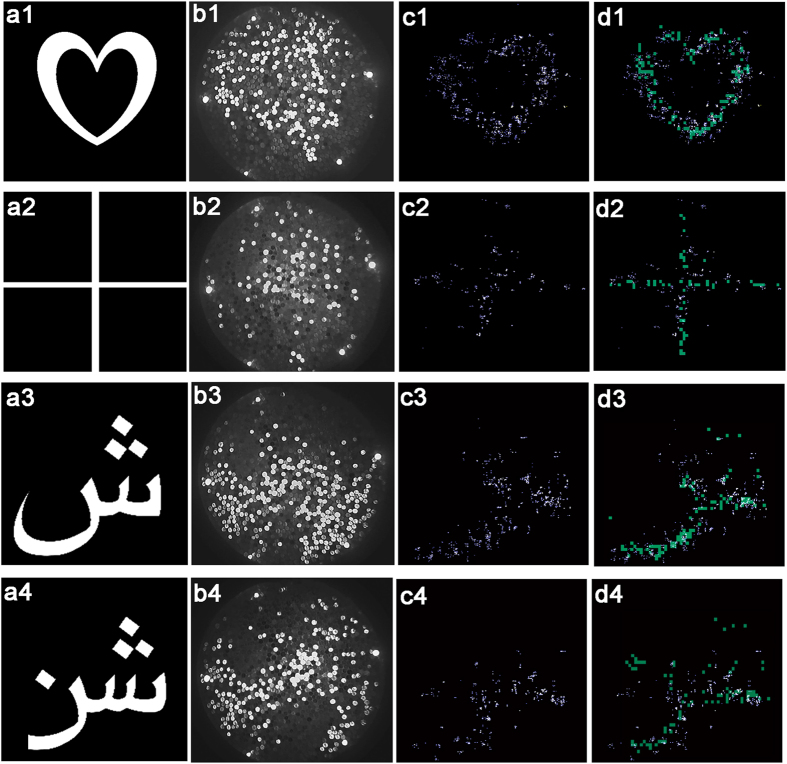
Measured results. Input images of (**a1**) a heart (**a2**) a cross (**a3**) aPersian letter “ش” and (**a4**), a Persian word “”. The images are fed into the fiber brush by a projector. (**b1**–**b4**) Shuffled images that is output from the brush (the images are 17 mm × 17 mm). (**c1**–**c4**) Deshuffled images of heart and cross based on off-axis ToF deshuffling technique (the images are 17 cm × 17 cm). The spots are weighed based on the original intensity of the fibers in the streak data cube—points with higher intensity represent fibers with brighter IR output from the sweeping pulses. (**d1**–**d4**) Deshuffled images are superimposed on coaxial raster scan reference (60 × 80 resolution) for comparison.

The ToF shuffling map is close to the raster reference (Fig. 3c1) but not identical. Quantitatively, the percentage of identically mapped fibers for both methods is a function of a similarity threshold that is represented by horizontal axis in Fig. 3c2. This threshold value is set on the difference between the address of the fibers found by raster and ToF technique. The ToF address values are mapped to raster address space (0–4800) to find a meaningful difference. After the difference value of 500 (shown by a white dashed line) the output of the raster and ToF method are over 90% alike, this value is just above 50% for address difference of 200. There are some notable outliers on the circumference, as the fibers on the edge can be sometimes too bright or too dim. Figure 4a1 also shows that the signal for initial fibers (in red and yellow) are much cleaner compared to that for the final fibers (mostly in blue) in the sweeping process. This is because the earlier fiber tips scatter and slightly distort the sweeping IR pulse for the later fibers. As in Fig. 3(a1) there is an exponentially decaying time-blur for each fiber too. This temporal blur can extend if thicker and longer fibers are used or if the fibers are submerged into a thick turbid medium. In our study we have used the peak intensity as the time of arrival; however, for longer time blurs temporal deconvolution would be necessary to correctly estimate the temporal signatures of each fiber. The acceptable time blur depends on the FWHM of the exponential decay relative to the entire time window. In our case with 1ns time window a FWHM of few hundreds of picoseconds is useable without deconvolution.

Finally, Fig. 4a1–a4 show four input scenes that are fed into the optical brush. As seen in Fig. 4b1–b4, the input is completely shuffled and some of the pixels are lost. Figure 4c1–c4 shows the deshuffled version of b1–b4 by ToF technique.

Figure 4d1–d4 shows ToF results superimposed on top of a lower resolution reference obtained by raster method (cyan color). As seen in Fig. 4d1–d4 the ToF results agree with the reference, with few outliers. Some broad-scale distortion (e.g. see the difference on the left edge of the heart in Fig. 4d1) is present due to the slight spherical wavefront curvature of the sweeping pulses at the open-end that is neglected in our calculations. The ToF technique provided 400 × 400 lateral resolution (X-Y) based on the time resolution of streak data cubes. The raster scan with our projector provided 60 × 80 resolution due to practical limitations, therefore, we had to up-sample the reference to properly compare the results (Fig. 4d1–d4).

Figure 4a3,a4 show results for two inputs with asymmetric and discontinuous outlines. The higher reconstruction error in the bottom left corner is because of lower intensity of the received light and aggregation of the error induced by the prior fibers closer to the illumination reference on the right (for X-scan) and on the top (for Y-scan).The quality of the reconstructed images should be compared to that obtained from raster scan as in Fig. 4d1–d4. The lower quality of images compared to a lens based image is the direct results of having a small number of fibers (only 1100 fibers or pixels total) and applying no prior in image reconstruction.

### Optical Brush Field of View

The geometric model of brush-like structures is well studied in computer graphics^37^. Here, however, we are trying to look at the physical properties of image acquisition with a brush-like form. The parameters of interest are the sampling pattern, the numerical aperture of each fiber, and the profile of Signal-to-Noise (SNR) with regards to these parameters.

**Figure 5:**
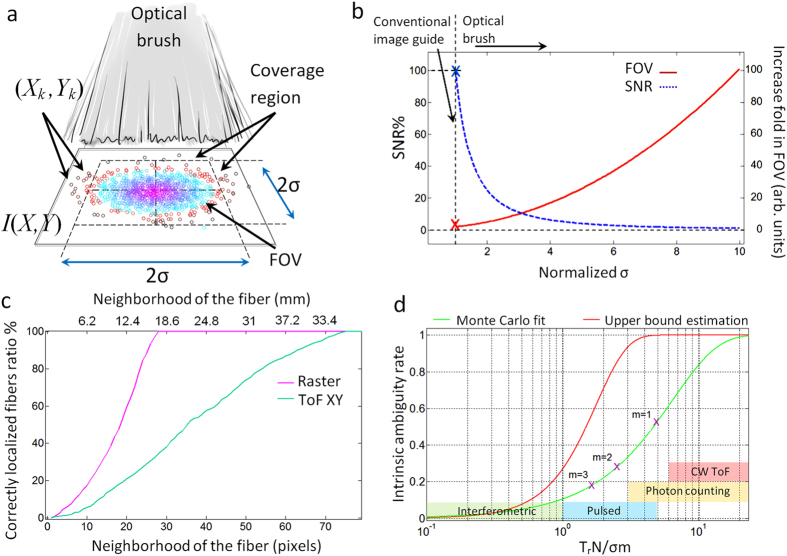
Brush form factor properties and localization accuracy. (**a**) Fibers spatial distribution shown by circular markers on the scene plane. The FOV is a function of fibers location distribution, the fibers that are too far apart from the center of the distribution cannot contribute to the signal (red circles) as the sampling rate gets lower and lower moving away from the center. A simplified square coverage region is approximated by FOV. (**b**) Dynamic of SNR, coverage region and 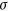. Blue dashed curve is the SNR and red solid curve shows coverage region. (**c**) Localization accuracy comparison of ToF and raster reference with the ground truth. (**d**) Ambiguity rates estimation based on MC simulation fit and an explicit upper bound estimation. Purple crosses show the ambiguity rate for the brush parameters used in our experiment for m = 1, 2, 3 from right to left respectively. Colored regions show the ambiguity rate range expected for different technologies based on their time resolution assuming a brush with 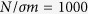.

We start with the assumption that the fiber tips’ coordinates (X and Y) are initially distributed in the scene with an independent and identically distributed (i.i.d.) normal distribution as 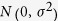 (Fig. 5a). As the fibers are now randomly placed in the scene the actual field of view (FOV) of such form factor for a single acquisition will be the area in which the image is resolvable with a desired SNR level. Therefore, the FOV is directly dependent on the average distance between the fibers (average spatial sampling rate) which itself is a function of number of fibers N and brush open-end distribution variance 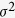. A universal and explicit formula for the exact FOV of the brush is beyond the scope of this work, as the image reconstruction quality can change based on many parameters such as prior knowledge of the scene, numerical aperture of each fiber, permutation of the bristles, inpainting technique, etc. Here we obtain an FOV estimation by considering a coverage region that contains most of the fibers but eliminates the fibers that are positioned too far off (further than an agreed threshold) from the center of the distribution.

Based on the normal distribution of two i.i.d. variables X and Y, a square with 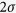 sides would contain 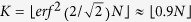 of the fibers in it. In this case, the average distance between the positions of the bristles can be found as 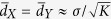 respectively. Considering the nonuniform version of Shanon sampling theorem (Whittaker–Shannon–Kotelnikov theorem) the condition; 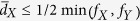 should hold for the scene to be fully recoverable. *f*_*x*_ and *f*_*y*_ are the highest horizontal and vertical frequencies of the 2D fourier transform of the scene. This only holds for the case in which all the samples are read without loss and noise. On the other hand, compressive sensing techniques demonstrate that prior knowledge of a scene can potentially relax this condition^38^.

If 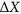 and 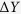 discretize the scene horizontally and vertically then the SNR at the back of the brush (considering the average coupling of the scene intensity into the brush) can be found as in equation (1),


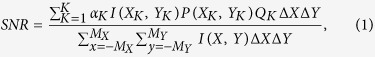


where, 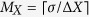 and 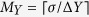 are horizontal and vertical resolutions, 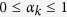 is the k^th^ fiber loss (this loss includes all the propagation, coupling-decoupling, and bending losses), and 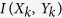 is the optical intensity of the scene at k^th^ fiber position 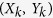. 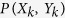 is the probability of the k^th^ fiber being located at 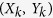 and being correctly registered (correctly deshuffled) at this location, *Q*_*k*_ is the number of pixels of the scene that is visible to k^th^ fiber. If fiber losses are identical 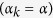 and the intensity profile is uniform 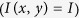 then a higher bound for equation (1) with *Q*_*k*_ = 1 can be found as:


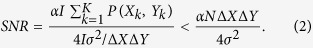


The numerator in equation (2) is proportional to the sum of fiber tips cross section and the denominator is the brush coverage area. Therefore, equation (2) indicates that with a fixed set of fibers (N) a larger coverage area is only obtainable at the price of a lower SNR (Fig. 5b). *Q*_*k*_ is a function of physical parameters such as numerical aperture and the distance of fiber tip to the scene plane. For instance, if the brush distance from a 2D Lambertian scene is increased; each fiber would see more pixels (receive light from more pixels) but consequently 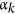 would be reduced as the intensity of the light that enters the fiber is reduced with distance. This parameter is considered *Q*_*k*_ = 1 for our experiment.

It must be noted that equation (1) and (2) both assume a direct multiplication of the scene image with the 2D spatial random distribution of the fibers (see matrix expression for image formation in supplementary note 1). This requires the fibers to be close to the scene or be fed with a projected image or a back reflected light. This might not always be the case (see supplementary note 1).

The chance of fibers being correctly deshuffled (the ratio of correctly localized fibers) depends on the neighborhood or error margin that is considered acceptable for the localization of each fiber. This is shown for both raster and ToF deshuffling methods in Fig. 5c. As expected, while lower in resolution, the coaxial raster reference is more accurate than ToF based technique. The pixel distance (horizontal axis) in this figure is the distance from the center of a fiber in a 3200 × 3200 ground truth image.

The deshuffling performance and thus the reconstruction error is affected by the average distance between the fibers relative to time resolution and dynamic range of the camera. Assuming the mentioned normal distribution for fiber tips locations, the intrinsic ambiguity between the fibers is estimated as in equation (3):


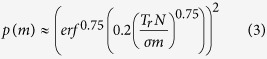


This estimation is obtained by fitting Monte Carlo simulations for intrinsic ambiguity rate among *m* neighboring fibers (see supplementary note 2). Here 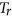 is the time resolution of the camera multiplied by speed of light in meters (depth resolution), 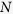 is the number of fibers, 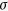 is the standard deviation of the normal distribution for the fibers and 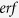
is the error function integral. The intrinsic ambiguity rate shows the fundamental ambiguity rate within a certain neighborhood that should be anticipated based on the randomness of the fibers positions. For example, for our study where 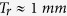, 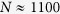, 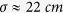, equation (3) indicates that 53% of the fibers have both X and Y coordinates below the depth resolution of the camera and thus cannot be distinguished with their closest neighboring fiber (Fig. 5d). This might seem as a large ambiguity rate; however, it must be noted that this error falls rapidly by increasing the neighborhood distance. For instance, the ambiguity rate falls to 28% and 18% for the second and third neighboring fibers respectively (purple crosses on green curve at Fig. 5d). Equation (3) also dictates the relative distribution of the brush and the proper technology that is going to work for that distribution. For example, for an optical brush with 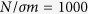 the horizontal axis of Fig. 5d would show the depth resolution in millimeters, and therefore, the range of anticipated ambiguity rate for different technologies can be found (this is shown as colored region in Fig. 5d)^19^,^24^,^28^,^29^. As indicated in Fig. 5d the CW ToF would have the highest ambiguity rate (almost 6 folds higher than pulsed mode) with the given brush parameters. This is anticipated as the time resolution (depth resolution) of such cameras is an order of magnitude coarser than streak-based systems. Therefore, for CW ToF the 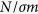 should be chosen in a way to reduce the intrinsic ambiguity rate. This means that either the number of the bristles N should be reduced or the brush should be further opened (increase in 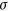) or the adjacent bristles should somehow be distinguished with the help of a secondary parameter (e.g. use of orthogonal temporal codes etc.).

The FOV of the brush should be covered by the time window (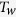) of the camera (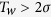) so that all the fibers are addressable. Also, the dynamic range of the camera can affect the performance if the sweeping X-scan, Y-scan pulses are not perfect plane waves. Since having a collimated beam at larger cross sections is impractical one should always consider the sweeping pulse intensity profile across the fibers. This intensity profile should fit into the dynamic range of the camera to avoid further ambiguity.

Our scene is approximately a patterned parallel beam bundle that is casted on the brush plane, therefore, the object distance to the brush is not a parameter of concern; however, a working distance could be defined and would be a parameter of significance if the fibers themselves were providing illumination to the scene or if a scattering scene was placed in front of the brush and illuminated with a secondary light similar to the case of a microscope (See supplementary note 3). It must be emphasized that the FOV of an optical brush is left intact as long as the signal received by each fiber is not changed. This makes an optical brush form factor very appealing for imaging or sensing in turbid or porous media because the opaque or diffuse space between the fiber tips does not directly affect the SNR. Similar to introduced optical brush, the lens-based endoscopes, also sacrifice SNR to gain a larger FOV, but they operate only in a transparent, uniform medium.

## Discussion

An optical brush is different from visual sensor networks in which different images of different cameras are used with homography techniques to reconstruct a 3D scene^39^. For an optical brush, it is the pixels or a small cluster of pixels that are shuffled and spread rather than full over lapping images as in the case of a visual sensor networks^15^,^39^.

The introduced off axis ToF-based deshuffling technique has the potential for real-time tracking of individual fibers in the medium. Ultimately the work can be extended to a fully self-calibrating brush, for which the fibers themselves bring the sweeping pulse and visible illumination to the scene and return the reflection signal from each fiber tip.

Nonuniform spatial sampling and low spatial resolution forced by the number of fibers are two of the limitations. Advanced localization methods or super resolution techniques might be of benefit to this end. Therefore, image inpainting methods and super resolution techniques can be used to recover the full original image by increasing the number of permutations and acquisitions. Both of these methods are well-studied topics in computer vision and are not the focus of this study. Additionally, multimode fibers don’t preserve coherency, therefore, interferometric approaches may be challenging for obtaining a more accurate localization. In order to improve the mapping results in our technique one can increase the number of references to get a better estimation of the location of the fiber. Also using a slightly higher power can enable the references to be further away to allow a wavefront with less curvature and less intensity variations.

The system demonstrated in this work does not have backlight for the fibers and treats each fiber as a single pixel. Additionally, there are only two scans that limit the localization to X and Y. By adding a 3rd “Z” scan, the full 3D localization of the fiber tips is possible. For more complex 3D geometries a more robust referencing (scanning) is necessary. For example, instead of only 2 or 3 reference points a fraction of the fibers could be used to time reference the rest of the fibers.

Single multimode fibers have been recently used as a medium for image transfer^40^. A combination of such techniques with optical brush can improve the deshuffling and resolution as the correlation and overlap between the images of each fiber can be exploited computationally.

Finally, we have demonstrated a proof of concept imaging through a large set of randomly distributed optical probes (an optical brush) based on an off-axis ToF calibration technique. This allows disconnected scene points to be imaged simultaneously via a large number of flexible optical fibers. Unlike the conventional imaging optics, the optical brush offers a flexible field of view that is tunable with redistribution of the bristles. This flexible form factor presents a new platform for probe-based imaging with large number of probes without the necessity for raster scanning or careful arrangement of the probes. The applications range from compressive sub-wavelength imaging to deformable optics for imaging in porous or turbid media and diffuse optical tomography^31^,^32^,^33^ with vast number of flexible probes.

## Methods

The brush 2D distribution on the open-end covers a circle with 20 cm diameter. The fibers are 25 cm long each. This is not necessarily an optimal length, it’s only chosen based on the field of view that we wanted to cover; longer fibers (4 to 5 times longer) can be used if necessary without significant increase in time blur. There is no mechanical scanning at the open-end of the brush, the scanning in X-Y domain occurs due to time difference of the reference pulse received by individual fibers as the pulse is propagating across the fiber ends. Since streak cameras provide only a 1D *x-t* slice we had to use a periscope mirror set (not shown in the schematics of Fig. 2a for simplicity) as in^21^ for sweeping the *y* dimension at the close-end of the fiber. The mechanical scanning time depends on the number of averaging that is needed for each x-t slice. This depends on the input power level of each fiber. In our experiment the full scan for each reference takes 20 minutes, therefore, it takes about 40 minutes to acquire both of the *x-y-t* data cubes for X reference and Y reference. While there is no fundamental limit for real-time operation our demonstration is for a single static random distribution of the brush bristles. If the random motion was involved then faster acquisition modalities such as use of single photon avalanche diodes could be considered to avoid motion artifacts. A more complex approach would be to feed all the fibers into a single streak image by compressive means.

For sorting purposes we used the maxima of the signal on each *x-t* column. The intensity of each point in the ToF based deshuffled image (Fig. 4c1–c4) is an integration of the intensity value of all the points that correlate with a time instance in the entire X-scan and Y-scan streak data cubes. This means that if both t_x_ and t_y_ are measured to be exactly the same within the time resolution of the system for more than one fiber (the fibers that are in ambiguity region), our algorithm will sum up the intensity of the measurements for those two fibers instead of overwriting or ignoring one of the fibers.

Feature-based image registration technique^41^,^42^ was used for registering the ToF data cubes with the ordinary camera image. To measure the ground truth position of the fibers on the open-end we used a 3200 × 3200 front image of the open-end with a DSLR camera as in Fig. 2b. It must be noted that the absolute number of the fibers that are localized via raster technique are smaller than that of ToF technique. Therefore, we used the ratio of the correctly localized fibers to compare the two techniques in Fig. 5c.

Fibers that are further away from the center axis of the brush (close to circumference) have a slight intensity drop because of the slight change in the angle of the fibers. Such nonuniform intensity output of fiber bundles is also noticed in previous studies with close-ended fiber bundles^4^,^13^. This nonuniformity can be compensated by either background subtraction or intensity normalization in consequent frames.

## Additional Information

**How to cite this article**: Heshmat, B. *et al.* Optical brush: Imaging through permuted probes. *Sci. Rep.* **6**, 20217; doi: 10.1038/srep20217 (2016).

## Supplementary Material

Supplementary Information

## Figures and Tables

**Figure 1:**
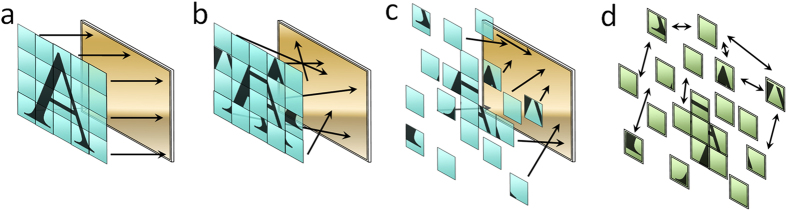
Scene points to sensor pixels mapping in different imaging modalities. (**a**) Coherent fiber bundle or an image guide coherently maps the scene (the letter “A”) onto the sensor. (**b**) Incoherent fiber bundle shuffles the pixels and feeds them to the sensor. (**c**) Optical brush randomly samples and shuffles the scene and then sends it to the sensor. (**d**) A hypothetical swarm optical sensor network can collectively image inside a medium, the pixels are communicating with each other.
