# Supplementary material for: Optical brush: Imaging through permuted probes
Source: Sci Rep. 2016 Feb 12;6:20217. doi: 10.1038/srep20217 (PMC4751542; doi:10.1038/srep20217)
Supplement: Supplementary Information [file srep20217-s1.pdf]

Optical brush: Imaging through permuted probes

Barmak Heshmat[1,*] Ik Hyun Lee[1], Ramesh Raskar[1]

[1]*Media Lab, Massachusetts Institute of Technology, 75 Amherst St., Cambridge, MA, 02139, USA*

[*]barmak@mit.edu

Supplementary Information

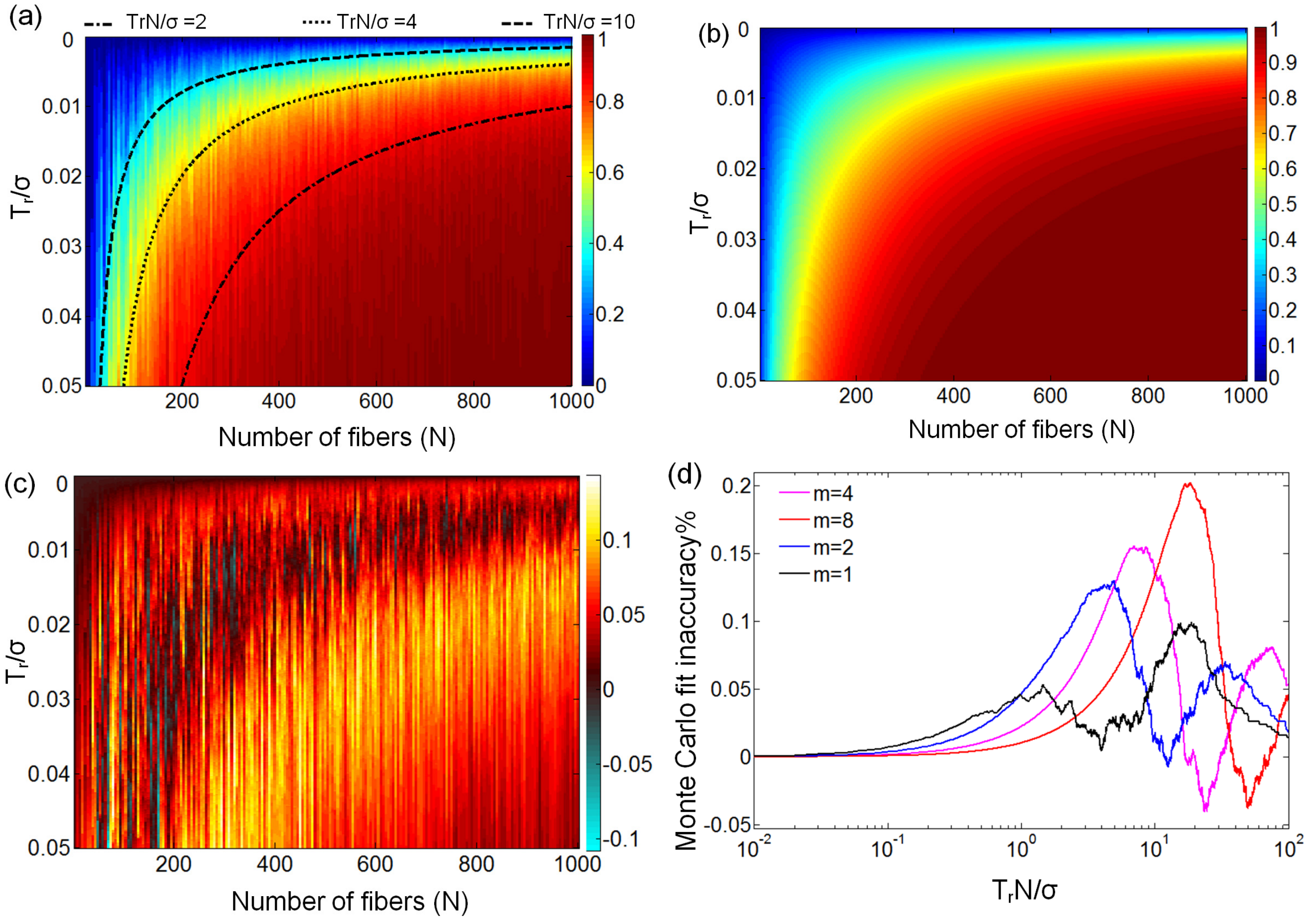

Supplementary Figure 1. a, Monte Carlo simulation of the ambiguity rate for first neighboring fiber. The ambiguity rate is constant for constant TrN/σ curve. **b,** MC simulation fit function as in supplementary equation (3). The fit function is from error function family.**c**, MC accuracy. The fit is fair within 15% accuracy. **d**, The fit function accuracy profile versus *m*. For each curve an MC simulation is updated and compared with the fit function as in Supplementary equation (3).

Supplementary Note 1 – Matrix expression for image formation with optical brush

Let I_{sc} be the vectorized scene intensity matrix with dimension $XY \times 1$ and I_{out} be the vectorized $N \times 1$ measured matrix at the end of the brush with the visible range camera; the matrix expression for an optical brush is given as:

$$I_{out} = P\left[\left(D * K_{er}\right) \circ I_{sc}\right], \quad (1)$$

where P is an $N \times XY$ permutation matrix with single nonzero entry in each row, D is the $XY \times 1$ distribution matrix which has a nonzero entry at the location of each fiber tip in the XY space, and finally, K_{er} is an $M \times 1$ kernel matrix where M is the size of the kernel. Here K is the number of fibers that is also equal to the number of ones in the permutation matrix. For matrix convolution, D is transformed into a $XY \times N$ Toeplitz matrix to perform convolution. The kernel matrix in our study is simply a single entry identity matrix since each fiber is coupled into a single pixel in the scene (Q_k=1). I_{out} is the output of the fibers that are vectorized based on their order in *xy* space.

By measuring the time-of-flight to localize each fiber at the XY space, we are indirectly measuring the permutation and distribution matrix. In other words $(p_k, p_{iX+j}) = (d_{iX+j}) = 1$ only if there is a pulse measured at $i = t_x$ and $j = t_y$. P_i and d_i are the *P* and *D* matrix entries. Additionally the *xy* visible range camera image is measuring the entry values of I_{out} for each fiber.

Supplementary Note 2 – Ambiguity rate expression and performance interpretation

The ambiguity rate is based on the normal independent identical distribution (i.i.d.) assumption for X_k and Y_k parameters. This rate is the fundamental ambiguity rate that is forced by the randomness of the positions. This is a notable rate since it predicts the highest bound on the performance (lowest error rate) of a brush system. To obtain this rate let's start with two fibers. By the normal i.i.d. and independent X_k and Y_k assumption for X and Y coordinates supplementary equation (2) can be derived. This is because a linear combination of i.i.d. normal variables is also a normal variable.

$$p(|X_2 - X_1| < T_r, |Y_2 - Y_1| < T_r) = p(-T_r < X_2 - X_1 < T_r)p(-T_r < Y_2 - Y_1 < T_r)$$
$$= \left[\int_{-Tr}^{Tr} \frac{1}{\sqrt{4\pi\sigma^2}} e^{-\frac{s^2}{4\sigma^2}} ds \right]^2 = erf^2(\frac{T_r}{2\sigma}) \tag{2}$$

Here T_r is the time resolution of the camera multiplied by speed of light in meters (depth resolution), t is an auxiliary integral variable, σ is the standard deviation of normal distribution for the fibers, and erf is the error function integral. By extending the same approach to all the fibers with positive X_k and Y_k supplementary equation (3) is obtained. The inequality in supplementary equation (3) is based on the symmetry of the normal distribution. An upper bound estimation on intrinsic ambiguity rate for the brush (*p(m)*) is thus estimated by finding the average distances for *m* neighboring fibers and finding the probability of that distance being less than depth resolution of the camera. This can be expressed as:

$$p(-T_r < m\frac{2}{N}\sum_{i=1}^{0.5N} X_k < T_r)p(-T_r < m\frac{2}{N}\sum_{i=1}^{0.5N} Y_k < T_r) < \left[\int_{-TrN/m}^{TrN/m} \frac{1}{\sqrt{4\pi\sigma^2}} e^{-\frac{s^2}{4\sigma^2}} ds \right]^2 = erf^2(\frac{T_r N}{2\sigma m}) < p(m), \tag{3}$$

where N is the total number of fibers. Supplementary equation (3) presents the following insights: first it indicates the analytic relation between fiber distribution parameters and camera parameters, second it predicts that the intrinsic ambiguity should decrease with increase in neighboring fibers, and third it indicates that the error should be of *erf* family. Despite the provided insight, supplementary equation (3) does not capture the fact that the distances are a nonuniform function of the fiber locations-

- this is important as it can induce a notable inaccuracy depending on the parameters. To calculate a more accurate bound on the ambiguity rate Monte Carlo simulations are necessary. Supplementary figure 1 a shows the results for such simulation, here we have generated a random set of variables with normal distribution and calculated the ambiguity rate numerically. The results immediately confirms that the ambiguity rate is constant on a $T_r N / \sigma = cte$ curve and thus it should be a function of it. Using insight from supplementary equation (3), we used erf-based function to fit the data. As seen in supplementary figure 1 c the Supplementary equation (4) seems to be a fair fit.

$$\left(erf^{0.75}\left(0.2\left(\frac{T_r N}{\sigma m} \right)^{0.75} \right) \right)^2 - p(m) \leq 0.15 \tag{4}$$

Supp. Fig 1 d shows the accuracy of this fit compared to Monte Carlo simulations with variation of m. A larger inaccuracy is induced by increase in the m number, however Supplementary equation (4) holds for $m<5$. This means that the fit is a good indication of the ambiguity rate of the fibers up to 5 neighboring fibers.

In case the positions were not independent and the X and Y positions had a joint Gaussian distribution, a multivariate Gaussian analysis should be used. Such analysis can be cumbersome as covariance matrix of all the fibers locations should be found. Therefore, a relaxing assumption such as i.i.d. variables can be very useful.

Supplementary Note 3 – Working distance of an optical brush

Since optical brush is not a lens based system the definition of "in focus image" and thus "working distance" is rather different for it. Unlike the case of lens-based systems where the sensor pixels are packed next to each other and the working distance is forced by the 3D point spread function (PSF) of the system. For an optical brush not only the PSF is probabilistic, but also the pixels are randomly positioned with large gaps in between them. The working distance, therefore, can be defined as the distance at which a certain percent (a%) of the illumination power from the FoV of a certain percent (b%) of the bristles is leaked into the mth adjacent fiber. In addition to these thresholds the optical brush working distance would be a function of $N / \sigma m$ parameter. Unlike a lens-based system where the depth of field starts far from the lens plane, for optical brush, the depth of field can start right from the brush plane and continue on up to the point where the shuffled output is considered out of focus based on the considered a, b, m parameters. For example, if a single point source is taken further and further away from the brush plane, at some distance more than one fiber can see that point and if taken even further, after a certain distance all the fibers would partially receive some illumination from that point source. In order to eliminate this complexity in our demonstration we have used synthetic 2D scene projected onto a brush plane which mimics the case where a scattering scene is in the working distance and thus in focus.
